# The N^6^-methyladenosine modification of circALG1 promotes the metastasis of colorectal cancer mediated by the miR-342-5p/PGF signalling pathway

**DOI:** 10.1186/s12943-022-01560-6

**Published:** 2022-03-19

**Authors:** Changwei Lin, Min Ma, Yi Zhang, Liang Li, Fei Long, Canbin Xie, Hua Xiao, Teng Liu, Buning Tian, Kaiyan Yang, Yihang Guo, Miao Chen, Jin Chou, Ni Gong, Xiaorong Li, Gui Hu

**Affiliations:** 1 Department of Gastrointestinal Surgery, The Third Xiangya Hospital of Central South University, Changsha, 410013 China; 2 Department of Hepatobiliary and Intestinal Surgery, Hunan Cancer Hospital and the Affiliated Cancer Hospital of Xiangya School of Medicine, Central South University, Changsha, 410013 China; 3 Hunan Chest Hospital, Changsha, 410013 China

**Keywords:** Colorectal cancer, Competitive endogenous RNA, N^6^-methyladenosine modification, circALG1, miR-342-5p, *Placental growth factor*

## Abstract

**Background:**

Previous studies have shown that the N^6^-methyladenosine (m^6^A) modification enhances the binding ability of mRNAs/long noncoding RNAs (lncRNAs) to microRNAs (miRNAs), but the impact of this modification on the competitive endogenous RNA (ceRNA) function of circular RNAs (circRNAs) is unclear.

**Methods:**

We used a human circRNA microarray to detect the expression profiles of circRNAs in 3 pairs of cancer and paracancerous tissues from patients with colorectal cancer (CRC) and 3 pairs of peripheral blood specimens from patients with CRC and healthy individuals. The circRNAs highly expressed in both peripheral blood and tumour tissues of patients with CRC, including circALG1, were screened. A quantitative reverse-transcription polymerase chain reaction (qRT-PCR) analysis of an expanded sample size was performed to detect the expression level of circALG1 in peripheral blood and tumour tissues of patients with CRC and determine its correlation with clinicopathological features, and circRNA loop-forming validation and stability assays were then conducted. Transwell assays and a nude mouse cancer metastasis model were used to study the function of circALG1 in CRC and the role of altered m^6^A modification levels on the regulation of circALG1 function. qRT-PCR, western blot (WB), Transwell, RNA-binding protein immunoprecipitation (RIP), RNA antisense purification (RAP), and dual-luciferase reporter gene assays were performed to analyse the ceRNA mechanism of circALG1 and the effect of the m^6^A modification of circALG1 on the ceRNA function of this circRNA.

**Results:**

CircALG1 was highly expressed in both the peripheral blood and tumour tissues of patients with CRC and was closely associated with CRC metastasis. CircALG1 overexpression promoted the migration and invasion of CRC cells, and circALG1 silencing and reduction of the circALG1 m^6^A modification level inhibited CRC cell migration and invasion. In vivo experiments further confirmed the prometastatic role of circALG1 in CRC. Further mechanistic studies showed that circALG1 upregulated the expression of *placental growth factor (PGF)* by binding to miR-342-5p and that m^6^A modification enhanced the binding of circALG1 to miR-342-5p and promoted its ceRNA function.

**Conclusion:**

M^6^A modification enhances the binding ability of circALG1 to miR-342-5p to promote the ceRNA function of circALG1, and circALG1 could be a potential therapeutic target in and a prognostic marker for CRC.

**Supplementary Information:**

The online version contains supplementary material available at 10.1186/s12943-022-01560-6.

## Background

Circular RNAs (circRNAs) are closed-loop noncoding RNAs lacking a 5′ cap structure and a poly (A) tail [1]. CircRNAs are not affected by RNA exonucleases, can stably exist in body fluids and are specifically expressed at different developmental stages, in different tissues and in different diseases, which makes them very promising diagnostic markers for diseases [2]. Jie Lin et al. [3] found that the expression levels of circRNAs in the plasma of patients with colorectal cancer (CRC) are significantly different from those in the plasma of healthy individuals, which indicates that circRNAs could be used as novel diagnostic markers for CRC. By studying the regulatory role of circRNAs in the occurrence and development of CRC, researchers have found that competitive endogenous RNAs (ceRNAs) constitute the main mode through which circRNAs exert their regulatory functions [4, 5]. Zeng et al. [6] found that circHIPK3 is highly expressed in CRC. Highly expressed circHIPK3 could competitively bind to miR-7, relieve the inhibitory effect of miR-7 on downstream target genes, and promote the proliferation, migration and invasion of CRC cells; therefore, this circRNA is a potential therapeutic target in CRC. However, the current research on circRNAs in CRC has significant limitations. First, circRNAs that serve as a diagnostic marker for CRC are often identified only from CRC tissues or peripheral blood specimens, and this screening fails to simultaneously consider both diagnostic markers for and therapeutic targets in CRC. Second, questions concerning the regulatory mechanism of ceRNAs in circRNAs remain unresolved; for example, whether other molecules are involved in the ceRNA process of circRNAs remains unclear.

N^6^-methyladenosine (m^6^A) modification is one of the most important modifications in RNA epitranscriptomics [7]. This modification plays an important role in the production and metabolism of RNA and affects the progression of a variety of diseases, including CRC [8]. Interestingly, recent studies have shown that m^6^A modification also plays an important role in ceRNA function [9, 10]. For example, Yang et al. [10] found that the m^6^A modification of lincRNA 1281 greatly enhances its binding ability to let-7. m^6^A modification is also widely present in circRNAs and plays an important role in the regulation of circRNA functions: it can regulate the nuclear export of circRNAs and affect the translation and stability of circRNAs [11]. Nevertheless, whether m^6^A modification regulates the ceRNA mechanism of circRNAs remains unclear.

Using microarrays, we screened circRNAs that could potentially be used as early diagnostic and therapeutic targets for CRC by analysing the expression profiles of circRNAs in CRC tissues and peripheral blood and explored the effect of the m^6^A modification of circRNAs on their ceRNA function.

## Materials and methods

### CircRNA microarray

To search for circRNAs that could be used as targets for the diagnosis and treatment of CRC, we collected 3 pairs of cancer and paracancerous tissues from patients with CRC and 3 pairs of peripheral blood specimens from patients with CRC and healthy individuals for the microarray detection of circRNAs. Total RNA from each sample was quantified using a NanoDrop ND-1000. Sample preparation and microarray hybridization were performed based on the standard protocols recommended by Arraystar. Briefly, total RNA was digested with RNase R (Epicentre, Inc.) for the removal of linear RNAs and enrichment of circRNAs. The enriched circRNAs were then amplified and transcribed into fluorescent cRNA utilizing a random priming method (Arraystar Super RNA Labelling Kit; Arraystar). The labelled circRNAs were hybridized onto the Arraystar Human circRNA Array V2 (8x15K, Arraystar). The slides were washed, and the arrays were scanned using an Agilent Scanner G2505C.

### Tissue specimens

Cancer and paracancerous tissues were collected from 40 patients with CRC who were pathologically diagnosed from January 1, 2019, to December 31, 2019, at Third Xiangya Hospital of Central South University, China. Blood specimens were also collected from 20 of these patients before and 1 week after surgery, and blood specimens from 15 healthy volunteers were collected from the Health Management Centre of the Third Xiangya Hospital of Central South University during the same period to serve as controls. The inclusion criteria for patients with CRC were as follows: (1) pathologically diagnosed with CRC; (2) received radical surgical treatment and had not received antitumour treatment before surgery; and (3) had complete pathological and clinical data. The paired cancer and paracancerous tissues were immediately stored in liquid nitrogen after tumour resection for the subsequent extraction of RNA and protein from the tissues. The collected clinical data included the sex, age, tumour size, tumour location, tumour differentiation degree, carcinoembryonic antigen (CEA), carbohydrate antigen 19–9 (CA19–9), tumour, node, and metastasis (TNM) staging.

### Cell culture

Normal human colon mucosal epithelial (FHC) cells and HT29, HCT116, SW480, and SW620 colon cancer cells were purchased from Nanjing KeyGen Biotech Co., Ltd., China. The FHC cells were cultured in RPMI 1640 medium (Gibco, USA) supplemented with 10% foetal bovine serum (FBS, Biological Industries, Israel) and penicillin/streptomycin solution. The HT29 and HCT116 cells were cultured in McCoy’s 5A medium (Keygen Biotech) supplemented with 10% FBS. The SW480 and SW620 cells were cultured in L15 medium (KeyGen Biotech) containing 10% FBS. All the cells were cultured in a cell culture incubator at 37 °C with 5% CO_2_. All the cells were identified within the past year based on short tandem repeats (STRs) or single nucleotide polymorphisms (SNPs) and were not contaminated with mycoplasma.

### Plasmid and lentivirus construction

The placental growth factor (PGF) overexpression plasmid, the dual-luciferase reporter gene plasmid, and the circALG1 overexpression and interference lentiviruses used in this study were purchased from Shanghai Genechem Co., Ltd. All the plasmids used were identified by DNA sequencing.

### Transfection

CRC cells were seeded in a 6-well plate and cultured for 24 h. Once the confluence reached 50–60%, Lipofectamine® 3000 was used for transfection following the procedure provided by the manufacturer. At a specific time after transfection, total RNA and total protein were extracted, and the transcription level of target genes and the protein expression of the proteins encoded by these genes were detected by quantitative reverse-transcription polymerase chain reaction (qRT-PCR) and western blot (WB), respectively.

### Lentiviral infection and screening of stably transfected cells

Lentiviral infection and screening of stably transfected cells were performed in accordance with a previously developed cell infection system [12]. Four days after infection, the intracellular fluorescence expression was assessed using an inverted microscope, and the medium was replaced with complete culture medium containing puromycin at a final concentration of 4 μg/mL. After continuous drug administration for no less than 5 days, qRT-PCR and WB assays were performed to detect the RNA levels of target gene and the levels of the proteins encoded by these genes. The infection efficiency was determined to screen for stably transfected cell lines.

### RNA extraction and qRT-PCR

Total RNA in cells and tissues was extracted in accordance with the manual provided with the TRIzol reagent (Thermo Fisher, USA). Total RNA in plasma was extracted using a BIOG plasma RNA extraction kit (Bio-generating Biotechnology Corp, China), and the extracted total RNA was treated with RNase-free DNase. The reverse transcription of mRNAs and circRNAs was performed in accordance with the manual provided with the ReverTra Ace qPCR RT Master Mix with gDNA Remover (Toyobo, Japan). Fluorescence quantitative PCR was performed in accordance with the manual provided with the KOD STBR® qPCR Mix reagent (Toyobo, Japan). The reverse transcription and fluorescence quantitative PCR of miRNAs were performed in accordance with the manual provided with the All-in-One™ miRNA qRT-PCR Detection Kit (GeneCopoeia, USA).

### Cell genomic DNA (gDNA) extraction and electrophoresis

Cell gDNA was extracted using an OMEGA Tissue DNA Kit (USA) in accordance with the manual provided with the kit. After successful gDNA extraction, agarose gel electrophoresis was performed in accordance with procedures described in the literature [13].

### WB

Total protein in cells or tissues was extracted using RIPA buffer and quantified using the BCA method. Subsequently, the proteins were electrophoresed on sodium dodecyl sulfate-polyacrylamide gel electrophoresis (SDS-PAGE) gels and transferred to polyvinylidene fluoride (PVDF) membranes. An enhanced chemiluminescence reagent kit was then used for detection.

### RNA FISH assay

RNA FISH was performed using an RNA FISH kit (Gene Pharma Co., Ltd.), and the probes used in this assay were ordered from Guangzhou RiboBio Co., Ltd. The specific procedure was performed in accordance with the manual provided with the kit. Briefly, the cells were plated in a small confocal dish and cultured overnight. After the supernatant was aspirated and discarded, the cells were fixed in paraformaldehyde and blocked in blocking solution, and the probe was hybridized overnight. The cells were then observed and photographed under a confocal fluorescence microscope.

### Dual-luciferase reporter gene assay

The dual-luciferase reporter assay was performed in accordance with the manual provided with the Dual Luciferase Reporter Assay Kit (Vazyme, China). First, the amount of plasmid needed for transfection was calculated. Cell lysis and fluorescence intensity assessments were performed 48 h after transfection.

### Transwell assay

Corning Transwell chambers 3422 and 354,480 were used for the assessment of migration and invasion, respectively, using the experimental procedure reported in the literature [12]. Briefly, after 24 h of starvation, the cells were seeded in a Transwell chamber (100,000/well). After 48 h of culture, the cells were fixed with methanol, stained with crystal violet, and observed and photographed under a microscope. The number of cells that crossed the membrane was determined.

### RNA immunoprecipitation (RIP) assay

A Magna RIP kit (Catalogue No. 17–700) was used in this study for the RIP assays. Briefly, SW480 cells were cultured in a 15-cm culture dish. The cells were fixed in accordance with the manual provided with the kit and lysed with lysis buffer containing protease inhibitors and RNase inhibitors. A coimmunoprecipitation assay was performed using an anti-m^6^A antibody or YTHDF1 antibody, and IgG was used as the isotype control. The RNA bound to the antibody-protein complex was then extracted using TRIzol reagent, and the expression of m^6^A-modified circALG1 was analysed by qRT-PCR.

### RNA antisense purification (RAP) assay

In this study, a BersinBio® RNA Antisense Purification (RAP) kit (catalogue no. Bes5103, BersinBio, China) was used. The specific procedure was performed in accordance with the manual. Briefly, after the target RNA was pulled down using a specific probe, other RNA and proteins that interacted with it were also adsorbed onto the magnetic beads. The products were then analysed by qRT-PCR and RAP-mass spectrometry (MS).

### Animal experiment

Six-week-old male BALB/c nude mice (body weight, 18–22 g) were used. All the animals were provided by the Department of Laboratory Animal Science at Central South University and were housed in a specific pathogen-free (SPF)-class experimental animal room to ensure an environment with 12 h of light and 12 h of darkness. Cells at the logarithmic phase of growth were collected and resuspended in phosphate buffered saline (PBS) to form a single-cell suspension. The cell density was adjusted to 1 × 10^7^/ml, and 200 μl of cell suspension was then injected via the tail vein. Starting from the day of cell inoculation, the growth of the 4 groups of nude mice was observed every 2 days, and the body weight of the animals was recorded and analysed. After 4 weeks of culture, an in vivo small animal imaging system was used to observe the metastasis of the tumour cells in the mice. The nude mice were sacrificed using the cervical dislocation method. The liver and lungs were rapidly removed with dissecting instruments and photographed. The removed tissues were fixed in 4% paraformaldehyde as soon as possible for subsequent haematoxylin and eosin (HE) staining. All animal procedures used in this study were approved by the Animal Ethics Committee of Central South University and conducted in accordance with the Guidelines for the Care and Use of Laboratory Animals.

### Ethics statement

This study was approved by the Ethics Review Committee of Third Xiangya Hospital of Central South University. All human tissue specimens were used with the informed consent of the patients and families, and the collection of these specimens was approved by the Ethics Committee of Third Xiangya Hospital of Central South University.

### Statistical analysis

GraphPad Prism 8.0 and SPSS 22.0 were used for data processing. The *t-*test and *χ*^*2*^ test were used to analyse the differences between different samples. A *p* value less than 0.05 was considered to indicate statistical significance (*p < 0.05, **p < 0.01, ***p < 0.001, ^#^*p* > 0.05).

## Results

### CircALG1 is highly expressed in CRC

**Fig. 1:**
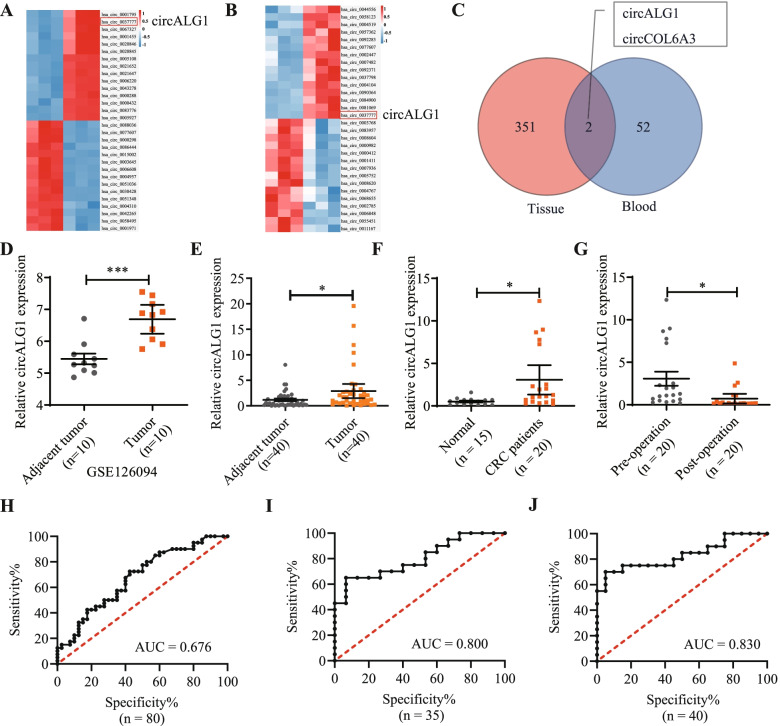
CircRNA expression profiling revealed that circALG1 is highly expressed in CRC. **A** Human circRNA microarray detection of circRNA expression in 3 pairs of cancer and paracancerous tissues from patients with CRC. **B** Human circRNA microarray detection of circRNA expression in 3 pairs of peripheral blood samples from normal individuals and patients with CRC. **C** Venn diagram of highly expressed circRNAs in both CRC tissues and peripheral blood. **D** Expression level of circALG1 in the GSE126094 dataset of the GEO. **E** qRT-PCR detection of the circALG1 expression levels in 40 pairs of CRC tumours and adjacent tissues. **F** qRT-PCR detection of the circALG1 expression levels in the peripheral blood of patients with CRC and normal individuals. **G** qRT-PCR detection of the circALG1 expression levels in the peripheral blood of patients with CRC before and 1 week after surgery. **H-J** *ROC* curves illustrating the discriminative capacity of circALG1 to distinguish between tumours and adjacent tissues from patients with CRC (**H**), between the peripheral blood of patients with CRC and that from healthy individuals (**I**), and between preoperative and postoperative peripheral blood samples from patients with CRC (**J**). The results are presented as the mean ± s.d. and are representative of at least 3 independent experiments. *p < 0.05, **p < 0.01, ***p < 0.001, ^#^*p* > 0.05

CircRNA microarray detection indicated that the expression levels of 353 circRNAs were significantly higher in cancer tissues from patients with CRC than in paracancerous tissues (Fig. 1A) and that the expression levels of 54 circRNAs were higher in the peripheral blood of patients with CRC than in that of healthy individuals (Fig. 1B). By analysing the circRNAs highly expressed in both CRC tissues and peripheral blood, we identified 2 circRNAs of interest, namely, circALG1 and circCOL6A3 (Fig. 1C). We then used the online website SRAMP (http://www.cuilab.cn/sramp) [14] to predict whether these 2 circRNAs might undergo m^6^A modification and found that both could experience this modification (Supplementary Fig. 1A). Because the overall expression level of circALG1 was higher than that of circCOL6A3 in the tissues and peripheral blood of patients with CRC, we selected circALG1 as the subsequent focus of study. In addition, we used the GSE126094 dataset in the Gene Expression Omnibus (GEO) for validation and found that the expression level of circALG1 in CRC cancer tissues was significantly higher than that in paracancerous tissues (Fig. 1D).

To further determine the expression level of circALG1 in CRC, we collected 40 pairs of cancer and paracancerous tissues from patients with CRC, preoperative and 1-week postoperative blood specimens from 20 patients with CRC, and peripheral blood specimens from 15 healthy individuals for qRT-PCR. The results indicated that circALG1 was highly expressed in both the cancer tissues (Fig. 1E) and peripheral blood (Fig. 1F) of patients with CRC. More interestingly, the expression level of circALG1 was significantly decreased in the peripheral blood of patients with CRC 1 week after surgery (Fig. 1G). Receiver operating characteristic (*ROC*) curve analysis showed that the expression level of circALG1 was well distinguished in the cancer and paracancerous tissues of patients with CRC (Fig. 1H), the peripheral blood of patients with CRC and healthy individuals (Fig. 1I), and the preoperative and postoperative peripheral blood of patients with CRC (Fig. 1J).

A correlation analysis of the circALG1 expression levels and clinical pathological parameters of patients with CRC indicated that patients with high circALG1 expression had higher levels of carbohydrate antigen 19–9 (CA19–9) and were more prone to metastasis (Table 1). Therefore, this study focused on the function of circALG1 in tumour metastasis.

**Table 1:** The correlation between circALG1 and clinicopathological features

	circALG1 high expression group	circALG1 low expression group	*P*
Sex			1.000
Male	11	11	
Female	9	9	
Age			0.058
≤ 60 years	13	7	
>60 years	7	13	
Smoking			0.077
No	19	15	
Yes	1	5	
Tumor location			0.342
Colon	9	12	
Rectum	11	8	
CEA			0.744
Normal	7	8	
High	13	12	
CA-199			**0.028**
Normal	18	12	
High	2	8	
Differentiated degree			0.465
High-middle	14	16	
Low	6	4	
T stage			1.000
1–2	1	1	
3–4	19	19	
LN metastatic^a^			0.519
No	13	11	
Yes	7	9	
M stage			**0.035**
0	20	16	
1	0	4	
TNM stage			0.337
I-II	13	10	
III-IV	7	10	

^a^ Lymph node metastatic

### Characteristics of circALG1 in CRC cells

**Fig. 2:**
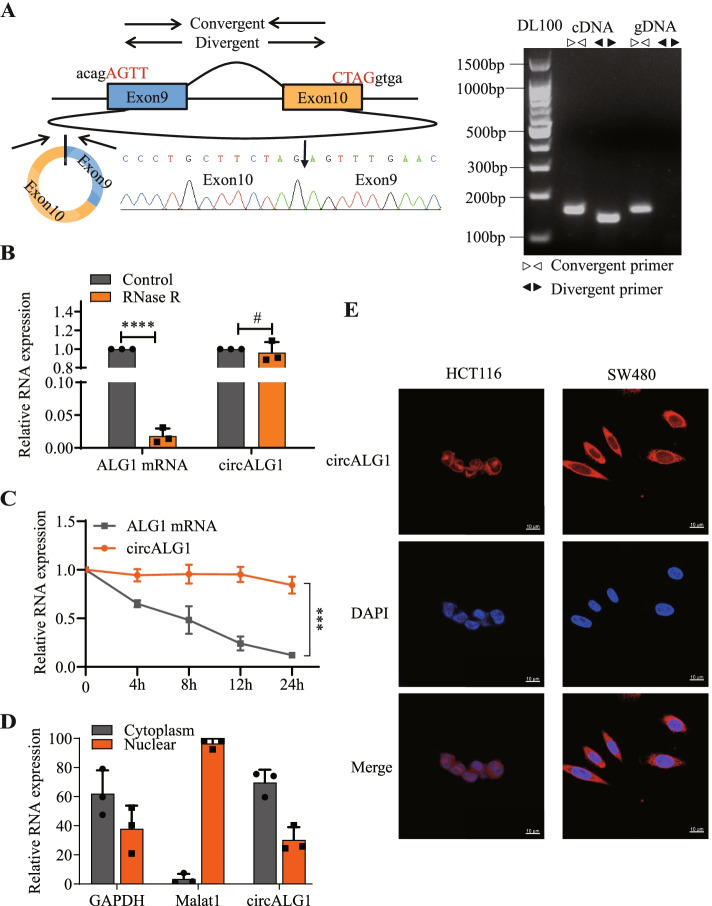
Characterization of circALG1 in CRC cells. **A** The expression of circALG1 was detected by qRT-PCR followed by Sanger sequencing. The arrows represent different primers (left). The RT-PCR products from different primers were detected by agarose gel electrophoresis (right). **B** SW480 cells were treated with RNase R, and the expression of circALG1 and its maternal gene ALG1 was then assessed by qRT-PCR. **C** SW480 cells were treated with actinomycin, and the stability of circALG1 was then assessed by qRT-PCR. **D** Analysis of the subcellular localization of circALG1 using a nucleus-cytoplasm separation assay. **E** Subcellular localization of circALG1 detected by FISH. The results are presented as the mean ± s.d. and are representative of at least 3 independent experiments. *p < 0.05, **p < 0.01, ***p < 0.001, ^#^*p* > 0.05

We designed a convergent primer and a divergent primer for the loop-forming sites of circALG1. Agarose gel electrophoresis showed that the convergent primer amplified bands in both cDNA and gDNA, whereas the divergent primer only amplified bands in cDNA. Further cDNA sequencing revealed the presence of circularization sites (Fig. 2A), indicating the circularity of circALG1. Compared with the parental gene ALG1 mRNA, circALG1 showed resistance to RNase R digestion (Fig. 2B). After actinomycin treatment, the degradation rate of circALG1 in cells was significantly slower than that of ALG1 mRNA (Fig. 2C), which indicated that circALG1 was stable. The nucleus-cytoplasm separation assay demonstrated that circALG1 was mainly localized in the cytoplasm (Fig. 2D). Similar results were obtained from circALG1 FISH (Fig. 2E).

### CircALG1 promotes CRC metastasis both in vitro and in vivo

We examined the expression levels of circALG1 in 5 cell lines, i.e., FHC, HT29, HCT116, SW480, and SW620. Compared with that in FHC cells, circALG1 expression was lower in HT29 and HCT116 cells and higher in SW480 and SW620 cells (Supplementary Fig. 1B). HCT116 cells, which exhibited the lowest circALG1 expression, and SW480 cells, which exhibited the highest circALG1 expression, were selected for subsequent functional experiments.

**Fig. 3:**
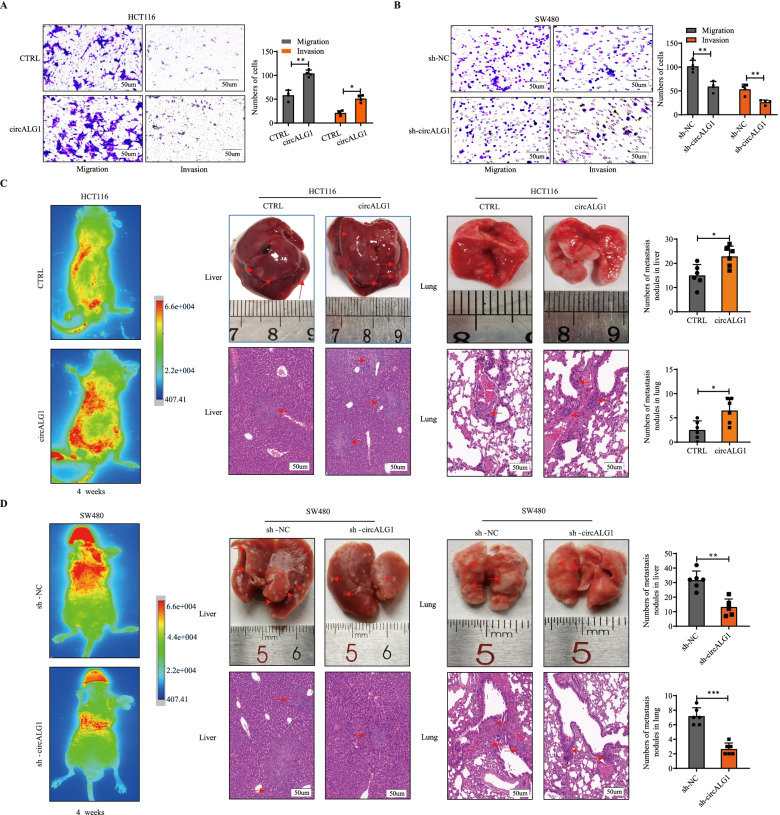
circALG1 promoted CRC metastasis in vivo. **A** Representative images and bar graphs of Transwell migration and invasion assays of HCT116 cells with or without circALG1 overexpression (**A**) and SW480 cells with or without circALG1 silencing (sh-circALG1) (**B**). **C-D** Representative images and bar graphs of liver and lung metastases with circALG1-overexpressing HCT116 cells (**C**) and circALG1-silenced SW480 cells (**D**) in a nude mouse metastatic tumour model. The results are presented as the mean ± s.d. and are representative of at least 3 independent experiments. *p < 0.05, **p < 0.01, ***p < 0.001, ^#^*p* > 0.05

We constructed stably transfected HCT116 cells that overexpressed circALG1, and the overexpression efficiency is shown in Supplementary Fig. 1C. We designed 3 siRNAs for circALG1 interference, and the knockdown efficiency is shown in Supplementary Fig. 1D. The si-3 sequence was used to construct a lentivirus that interfered with circALG1 expression, and a stably transfected cell line was generated. The Transwell assay results demonstrated that the migration and invasion abilities of HCT116 cells were enhanced after circALG1 overexpression (Fig. 3A), whereas the migration and invasion abilities of SW480 cells were weakened after the silencing of circALG1 expression (Fig. 3B).

Then, we tested whether the parental linear ALG1 could affect the function of circALG1. qRT-PCR showed that the overexpression and interference of circALG1 had no effect on the expression of linear ALG1 mRNA (Supplementary Fig. 2A). We also constructed an ALG1 overexpression plasmid and transfected it into HCT116 and SW480 cells. The overexpression efficiency is shown in Supplementary Fig. 2B. We designed 3 siRNAs for ALG1 interference, and the knockdown efficiency is shown in Supplementary Fig. 2B. Transwell assays showed that ALG1 was not associated with any significant change in migration or invasion of HCT116 and SW480 cells (Supplementary Fig. 2C). These results showed that parental linear ALG1 did not contribute to the function of circALG1.

In the HCT116 nude mouse metastatic tumour model, higher numbers of liver and lung metastases were observed with the overexpression of circALG1 (Fig. 3C); and in the SW480 nude mouse metastatic tumour model, interference with circALG1 expression decreased the numbers of liver and lung metastases (Fig. 3D). These results indicated that circALG1 was closely related to CRC metastasis.

### CircALG1 m^6^A modification enhances the migration and invasion abilities of CRC

**Fig. 4:**
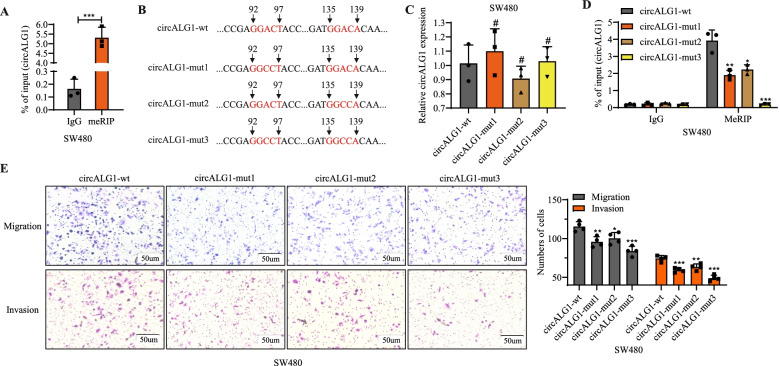
The m^6^A modification of circALG1 promoted CRC metastasis in SW480 cells. **A** MeRIP assays of m^6^A-modified circALG1 in SW480 cells. **B-D** Construction of an overexpression plasmid targeting the mutations at m^6^A modification sites of circALG1 (**B**), qRT-PCR detection of the circALG1 expression level (**C**), and MeRIP analysis of m^6^A-modification level of circALG1 (**D**). **E** Representative images and bar graphs of Transwell migration and invasion assays of cells with different circALG1 m^6^A modification levels. The results are presented as the mean ± s.d. and are representative of at least 3 independent experiments. *p < 0.05, **p < 0.01, ***p < 0.001, ^#^*p* > 0.05

Using the online website SRAMP, we found that circALG1 was highly likely to undergo m^6^A modification; additionally, circALG1 enrichment was observed in the methylated RNA immunoprecipitation (MeRIP) assay, which confirmed that circALG1 indeed underwent m^6^A modification (Fig. 4A). We studied the effect of the circALG1 m^6^A modification level on circALG1 function: after mutation of the 2 possible m^6^A modification sites in circALG1, wild-type and mutant plasmids were constructed (Fig. 4B). The same amount of wild-type and mutant plasmids was used to transfect SW480 cell lines in which circALG1 was stably silenced. A qRT-PCR analysis indicated no difference in the expression levels of circALG1 among the different groups (Fig. 4C); the intracellular levels of m^6^A-modified circALG1 were reduced when 1 site was mutated, whereas no intracellular m^6^A-modified circALG1 was observed when both sites were mutated (Fig. 4D). Functional experiments showed that circALG1 mutation decreased the migration and invasion of tumour cells (Fig. 4E). Similar results were also observed in HCT116 cells (Supplementary Fig. 3). These results indicated that the level of circALG1 m^6^A modification was positively correlated with the migration and invasion abilities of CRC cells.

### CircALG1 enhances the migration and invasion of CRC cells by acting as a miR-342-5p sponge

CircRNAs exert their functions through many mechanisms. The most common mechanism is ceRNA; that is, by competitively binding miRNAs, the inhibitory effect of miRNAs on target gene mRNAs is relieved, and this process is performed in the cytoplasm [15]. Previous studies revealed that circALG1 is mainly localized in the cytoplasm, which indicates that circALG1 may mediate CRC metastasis through ceRNA. We used Arraystar miRNA target prediction software (Arraystar, Rockville, MD, USA) [16, 17] to predict miRNA response elements (MREs) and obtained 5 miRNAs that were most likely to bind to circALG1: miR-302d-3p, miR-342-5p, miR-372-3p, miR-483-5p, and miR-520d-3p.

**Fig. 5:**
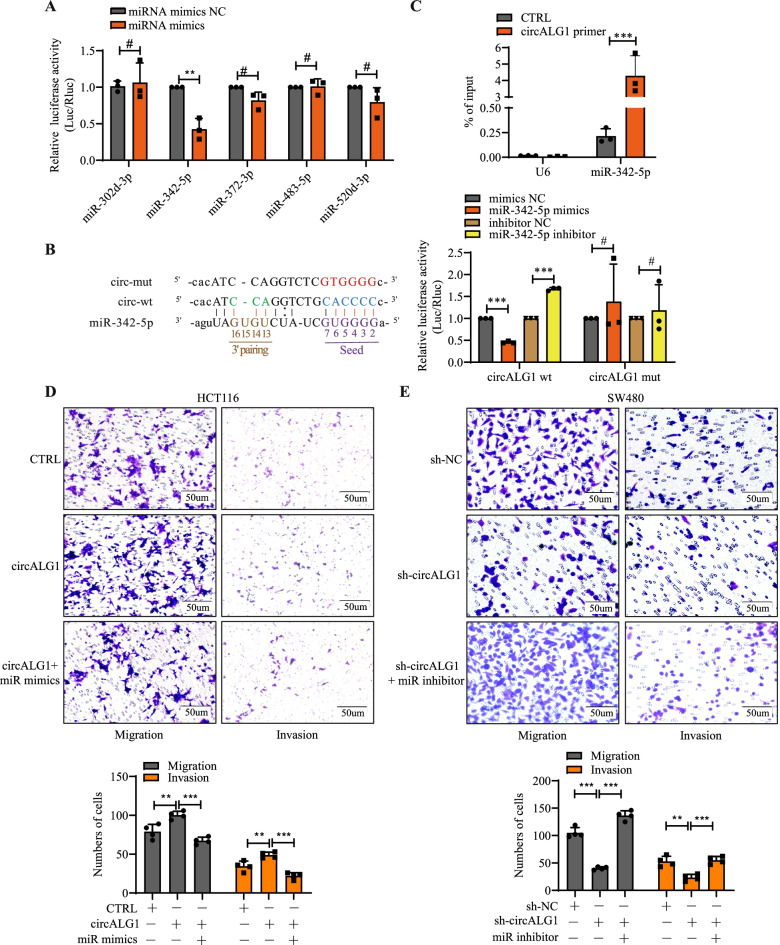
CircALG1 promoted migration and invasion through miR-342-5p in CRC. **A** Luciferase expression was normalized to Renilla luciferase expression, and the relative luciferase activity of miRNA mimic-transfected wells was compared with the control luciferase levels. **B** The luciferase activities were measured by a dual-luciferase assay, and the Renilla/firefly luciferase light-unit ratio was calculated. **C** qRT-PCR detection of miR-342-5p enrichment by circALG1 RAP assay. **D-E** Representative images and bar graphs of Transwell migration and invasion assays of cells with circALG1 overexpression and treatment with the miR-342-5p inhibitor (**D**) and cells with circALG1 inhibition and treatment with miR-342-5p mimics (**E**). The results are presented as the mean ± s.d. and are representative of at least 3 independent experiments. *p < 0.05, **p < 0.01, ***p < 0.001, ^#^*p* > 0.05

To study the interaction between these 5 miRNAs and circALG1, we conducted the following experiment. The SV40-firefly_Luciferase-MCS plasmid containing the circALG1 wild-type sequence was constructed and cotransfected with miRNA mimics (Supplementary Fig. 4A) and a Renilla luciferase plasmid. A decrease in fluorescence intensity was observed in the miR-342-5p group (Fig. 5A), which suggested that miR-342-5p might bind to circALG1. We then assessed the expression level of miR-342-5p in CRC and found that miR-342-5p expression was low in CRC tissues (Supplementary Fig. 4B). Based on the predicted binding sites of circALG1 and miR-342-5p, we designed an SV40-firefly_Luciferase-MCS plasmid containing the circALG1 mutant sequence (Fig. 5B, left). The dual-luciferase reporter assay results indicated that after transfection of the firefly luciferase plasmid containing the circALG1 wild-type sequence, miR-342-5p mimics significantly reduced the fluorescence intensity, whereas the miR-342-5p inhibitor enhanced the fluorescence intensity. No difference was detected with the plasmid containing the circALG1 mutant sequence (Fig. 5B, right, Supplementary Fig. 4C), which indicated that miR-342-5p specifically bound to circALG1.

In the circALG1 RAP assay, we also observed miR-342-5p enrichment (Fig. 5C). Functional experiments showed that in HCT116 cells, miR-342-5p overexpression reversed the enhanced migration and invasion abilities induced by circALG1 overexpression (Fig. 5D), whereas in SW480 cells, miR-342-5p inhibition reversed the reductions in migration and invasion abilities induced by circALG1 silencing (Fig. 5E).

### PGF is a downstream target of the circALG1/miR-342-5p signalling axis

**Fig. 6:**
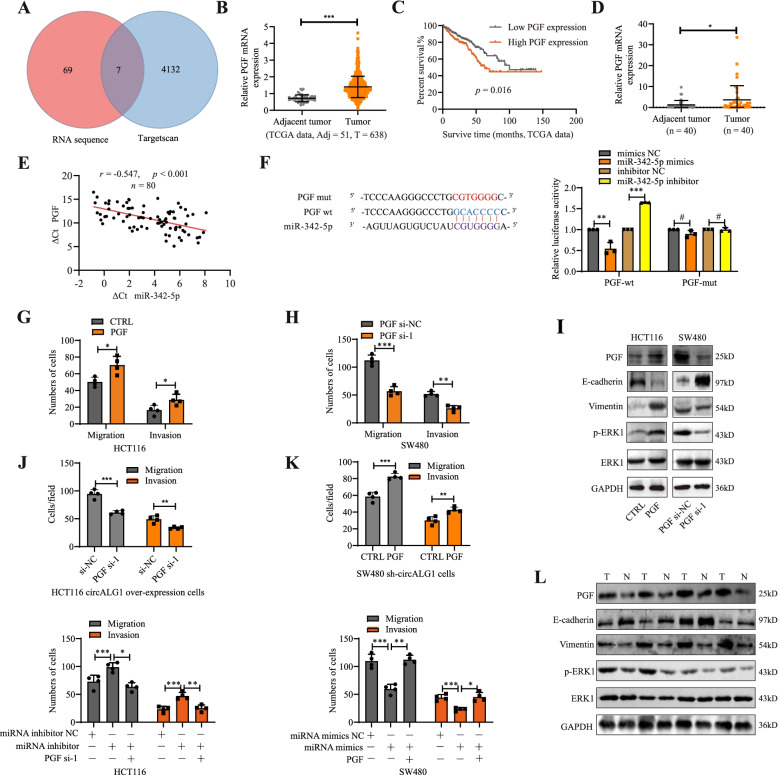
PGF was a downstream target of the circALG1/miR-342-5p signalling axis. **A** Venn diagram of RNA sequencing and downstream target genes of miR-342-5p predicted by TargetScan. **B** The expression level of PGF in TCGA COAD+READ data. **C** Prognostic analysis of CRC with low and high PGF expression. **D** qRT-PCR detection of PGF expression in tumours and adjacent tissues from patients with CRC. **E** The correlation between PGF expression and miR-342-5p was evaluated by Pearson’s correlation analysis. **F** The luciferase activities were measured by a dual-luciferase assay, and the Renilla/firefly luciferase light-unit ratio was calculated. **G-H** Bar graphs of Transwell migration and invasion assays of cells with different PGF expression levels*.* **I** WB analyses of total ERK and p-ERK in the MAPK signalling pathway as well as the EMT-related proteins E-cadherin and vimentin in cells with different PGF expression levels. **J-K** Bar graphs of Transwell migration and invasion assays of cells with PGF inhibition and treatment with the circALG1 overexpression plasmid (top, J) or miR-342-5p inhibitor (bottom, J) and cells with PGF overexpression and treatment with the circALG1 inhibitor (top, K) or miR-342-5p mimics (bottom, K). **L** WB detection of the expression levels of PGF, total ERK and p-ERK (MAPK signalling pathway) and E-cadherin and vimentin (EMT-related proteins) in cancer and paraneoplastic tissues from patients with CRC. The results are presented as the mean ± s.d. and are representative of at least 3 independent experiments. *p < 0.05, **p < 0.01, ***p < 0.001, ^#^*p* > 0.05

To determine the downstream target genes of the circALG1/miR-342-5p signalling axis, we extracted RNA from SW480 cells before and after circALG1 silencing for RNA sequencing. A total of 76 genes with > 2-fold upregulated expression levels were obtained. GO analysis of these 76 genes also showed that they were enriched in pathways related to metastasis (Supplementary Fig. 4D). a total of 7 genes were identified as miR-342-5p downstream target genes, as predicted by the TargetScan website and RNA sequencing (Fig. 6A). Through a search of the bioinformatics website UALCAN (http://ualcan.path.uab.edu/) [18, 19], we found that only PGF was highly expressed in CRC and suggested a poor prognosis (Fig. 6B-C, PGF was also highly expressed in the CRC specimens collected in this study (Fig. 6D).

A correlation analysis showed that the expression level of PGF in CRC tissues was negatively correlated with miR-342-5p (*R* = − 0.547, *p* < 0.001) (Fig. 6E). The dual-luciferase reporter assay results indicated that after transfection of the firefly luciferase plasmid containing the wild-type sequence of the *PGF* 3’UTR, miR-342-5p mimics reduced the fluorescence intensity, whereas the miR-342-5p inhibitor increased the fluorescence intensity. No similar phenomenon was observed after transfection of the firefly luciferase plasmid containing the mutant sequence of the PGF 3’UTR, which indicated that miR-342-5p could specifically bind to the PGF 3’UTR and inhibit its expression (Fig. 6F).

Compared with that in FHC cells, PGF expression was lower in HT29 and HCT116 cells and higher in SW480 and SW620 cells, which was consistent with the expression trend found for circALG1 in cells (Supplementary Fig. 4E). The efficiencies of PGF overexpression (Supplementary Fig. 4F) and silencing (Supplementary Fig. 4G) are shown in Supplementary Fig. 3. Ultimately, the PGF si-1 sequence was selected for functional experiments. PGF overexpression in HCT116 cells enhanced the migration and invasion abilities of the cells (Fig. 6G, Supplementary Fig. 5A), and in SW480 cells, PGF silencing reduced the migration and invasion abilities of the cells (Fig. 6H, Supplementary Fig. 5B). Previous studies have shown that PGF can activate tyrosine kinase receptors, promote extracellular signal-regulated kinase (ERK) phosphorylation, and activate the mitogen-activated protein kinase (MAPK) signalling pathway [20, 21]. The MAPK signalling pathway regulates many physiological activities, including inflammation, apoptosis, carcinogenesis, tumour cell invasion and metastasis [22, 23]. In this study, PGF overexpression significantly upregulated p-ERK level in CRC cells and reduced and increased the expression of the epithelial-mesenchymal transition (EMT)-related markers E-cadherin and vimentin, respectively; the opposite findings were observed after PGF silencing (Fig. 6 I). Therefore, PGF affects CRC metastasis by regulating EMT.

In addition, PGF silencing in HCT116 cells reversed the migration and invasion enhancement induced by circALG1 overexpression or miR-342-5p inhibition (Fig. 6J, Supplementary Fig. 5C-D), and in SW480 cells, PGF overexpression reversed the weakening of the migration and invasion abilities of cells caused by circALG1 inhibitor or miR-342-5p mimics (Fig. 6K, Supplementary Fig. 5C/E). These results confirmed the contribution of the circALG1/mir342-5p/PGF axis in CRC metastasis.

In CRC specimens, we also observed high PGF expression, increased p-ERK level and vimentin expression, and decreased E-cadherin expression (Fig. 6L). These results indicated that PGF was a downstream target gene of the circALG1/miR-342-5p signalling axis.

### The m^6^A modification of circALG1 enhanced its binding ability to miR-342-5p

**Fig. 7:**
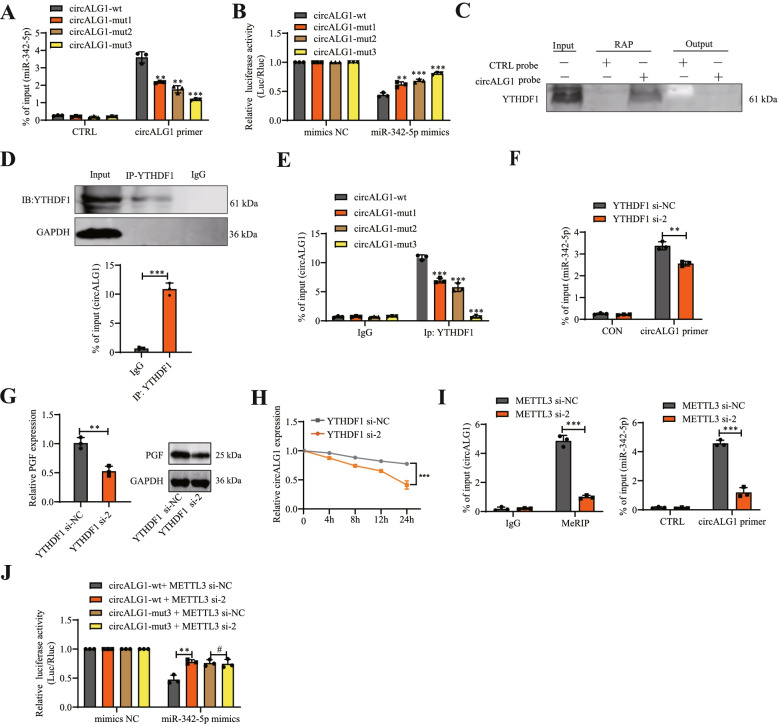
The m^6^A modification of circALG1 enhanced its ability to bind to miR-342-5p. **A** CircALG1 RAP detection of the enrichment of miR-342-5p in cells with different m^6^A modification levels. **B** The luciferase activities were measured by a dual-luciferase assay, and the Renilla/firefly luciferase light-unit ratio was calculated. **C** CircALG1 RAP proteins were evaluated by WB with YTHDF1 antibody**. D** qRT-PCR detection of the enrichment of circALG1 in the YTHDF1 immunoprecipitate obtained by RIP. **E** A RIP assay was used to detect the enrichment of circALG1 after mutation of the m^6^A modification site. **F** A RAP assay was used to detect the enrichment of miR-342-5p after YTHDF1 interference. **G** qRT-PCR and WB assays were performed to detect PGF expression levels after YTHDF1 interference. **H** CircALG1 stability in YTHDF1 si-NC/si-2 SW480 cells was determined by qRT-PCR after actinomycin D treatment for the time indicated. **I** MeRIP analysis of the circALG1 m^6^A modification levels (left) and miR-342-5p enrichment after METTL3 interference, as detected by circALG1 RAP (right). **J** The luciferase activities were measured by a dual-luciferase assay, and the Renilla/firefly luciferase light-unit ratio was calculated. The results are presented as the mean ± s.d. and are representative of at least 3 independent experiments. *p < 0.05, **p < 0.01, ***p < 0.001, ^#^*p* > 0.05

To study the effect of m^6^A modification on circALG1 function, we performed a circALG1 RAP assay using SW480 cells in which circALG1 was stably silenced and transfected with the constructed wild-type and m^6^A-modified mutant circALG1 plasmids. The results showed that a reduction in the m^6^A modification of circALG1 attenuated its binding to miR-342-5p (Fig. 7A). We also constructed corresponding firefly luciferase plasmids based on the wild-type and m^6^A-modified mutant circALG1 sequences. A dual-luciferase reporter assay indicated that mutation of the circALG1 m^6^A modification site enhanced the luciferase expression and increased the fluorescence intensity, and the highest fluorescence intensity was observed when both m^6^A modification sites were mutated. These results indicated that the inhibitory effect of miR-342-5p mimics on the expression of the firefly luciferase plasmid containing the circALG1 with the m^6^A modification site mutated sequence was attenuated, which suggested that the binding of circALG1 to miR-342-5p was regulated by m^6^A modification (Fig. 7B).

We then investigated the specific mechanism through which m^6^A modification affects the binding of circALG1 to miR-342-5p. The literature suggests that the function of circRNA m^6^A modification often requires the involvement of m^6^A modification-related proteins [24–26]. MS detection of the proteins pulled down in the circALG1 RAP assay showed that YTHDF1 enrichment was highest among the m^6^A modification-related proteins; therefore, this protein was preliminarily selected as the focus of study. The presence of YTHDF1 was also observed among the pulled-down proteins (Fig. 7C). Additionally, we detected circALG1 enrichment in the YTHDF1 RIP assay (Fig. 7D). When the circALG1 m^6^A modification site was mutated, the YTHDF1 RIP assay results indicated that the enriched circALG1 was significantly reduced, which suggested that the binding of circALG1 to YTHDF1 was dependent on m^6^A modification (Fig. 7E). To further study the effect of YTHDF1 on circALG1 function, we designed siRNA targeting YTHDF1, and the siRNA interference efficiency is shown in Supplementary Fig. 6A. The results showed that the downregulation of YTHDF1 expression reduced the binding ability of circALG1 to miR-342-5p (Fig. 7F) and decreased the expression level of the target gene, i.e., PGF, in the circALG1/miR-342-5p signalling axis (Fig. 7G) through the specific mechanism of YTHDF1 enhancing the stability of circALG1 (Fig. 7H).

We also studied the changes in ceRNA function after circALG1 m^6^A modification from the perspective of the m^6^A writer. The circALG1 m^6^A motif is RRACH (*R* = A or G; H = A, C or U), which can be read by METTL3 and catalyse the methylation of adenylate. We designed METTL3 siRNAs for related functional experiments, and the METTL3 knockdown efficiency is shown in Supplementary Fig. 6B. The silencing of METTL3 reduced the level of circALG1 m^6^A modification and weakened the binding ability to miR-342-5p (Fig. 7I). With the firefly luciferase plasmid containing the circALG1 wild-type sequence, the silencing of METTL3 weakened the inhibition of luciferase plasmid expression by miR-342-5p and increased the expression of luciferase, which resulted in enhanced fluorescence. With the firefly luciferase plasmid containing the circALG1-mut3 sequence, no effect on the fluorescence intensity was observed after METTL3 silencing due to the lack of an m^6^A modification site (Figs. 7J ). This result also suggested that m^6^A modification could enhance the binding ability of circALG1 to miR-342-5p.

Furthermore, we found that the stability of circALG1 decreases with METTL3 interference due to downregulation of the m6A modification level of circALG1 (Supplementary Fig. 6C). We further detected the RNA of MeRIP assay pull-down and found the presence of ALG1 pre-mRNA. Interestingly, after METTL3 interference, the m6A modification level of ALG1 pre-mRNA also decreased (Supplementary Fig. 6D), and the expression of circALG1 was downregulated (Supplementary Fig. 6E). These results indicate that m6A modification of ALG1 pre-mRNA may promote circALG1 splicing and thus upregulation of circALG1 expression. Of course, further investigation is required.

## Discussion

In recent years, the important regulatory role of circRNAs, as noncoding RNAs, in tumour occurrence, development and metastasis has received increasing attention from researchers [27, 28]. In this study, we found that circALG1, which is highly expressed in CRC, promoted CRC metastasis through the circALG1/miR-342-5p/PGF signalling axis and that m^6^A modification enhanced the binding ability of circALG1 to miR-342-5p and promoted the ceRNA mechanism.

CircRNAs are highly evolutionarily conserved, and their distribution exhibits tissue, timing, and disease specificity [29]. Many studies have shown that circRNAs have value in disease diagnosis and efficacy evaluations [2, 30, 31]. For example, circCDYL can be used as a potential prognostic predictor for patients with breast cancer [32]. In this study, circALG1 was screened through a circRNA microarray analysis of peripheral blood and tissue samples from patients with CRC. This circRNA is derived from 2 exons (exons 9 and 10) of the gene encoding ALG1. To date, no studies related to circALG1 have been published. Additionally, the parental gene ALG1 has not been reported to be involved in cancer. We found that circALG1 was highly expressed in cancer tissues from patients with CRC and was more highly expressed in the peripheral blood from patients with CRC than in that of healthy individuals. Interestingly, we also found that after radical tumour resection, the expression level of circALG1 in the peripheral blood from patients with CRC decreased, which suggested that circALG1 might be a potential indicator for the diagnosis of CRC and the evaluation of postoperative efficacy. Therefore, we plan to confirm its potential clinical significance in subsequent studies using a large number of clinical samples.

In terms of function, different subcellular localizations of circRNAs determine their functions. Among them, circRNAs located in the nucleus mainly regulate the transcriptional expression of genes, alternative gene splicing, and epigenetic modifications, whereas circRNAs localized in the cytoplasm can function as miRNA molecular sponges or encode polypeptides [33]. In this study, we found that circALG1 is mainly localized in the cytoplasm and can function through the ceRNA mechanism, and parental linear ALG1 had no functional effect on circALG1. Further bioinformatics analysis and dual-luciferase reporter gene assays showed that circALG1 competitively adsorbed miR-342-5p to relieve the inhibitory effect on the target gene *PGF* and thereby promote CRC metastasis. As a growth factor, the target gene *PGF* can activate the MAPK signalling pathway and promote ERK activation. As a stabilizing factor of mesenchymal transcription factors, p-ERK can inhibit the degradation of mesenchymal transcription factors such as ZeB, Snail, Slug, and Twist, which results in loss of the epithelial characteristics of tumour cells [34–36]. In this study, we observed that the upregulation of PGF expression increased the degree of ERK protein phosphorylation reduced the expression of the EMT-related protein E-cadherin and increased the expression of vimentin.

M^6^A modification, which is the most common epigenetic modification of RNA, affects RNA precursor shearing, regulates the nuclear export of RNA, promotes RNA translation, affects RNA stability, and enhances the association with miRNA [7]. This modification is also common in circRNAs. Functionally, the m^6^A modification of circRNAs can regulate the eukaryotic transcriptome and affect the splicing, export, localization, translation, and stability of circRNAs [11]. Although m^6^A modification enhances the ability of mRNAs and lincRNAs to bind miRNAs and thereby affects ceRNA mechanisms, this phenomenon has not been reported in circRNAs. We found that m^6^A modification promoted the binding of circALG1 to miR-342-5p and enhanced the function of circALG1 ceRNA and that this biological effect was mediated by YTHDF1. YTHDF1 is a member of the m^6^A RNA methylation recognition protein family, which also includes YTHDF2, YTHDF3 and YTHDC1. Different YTH proteins can exert different effects after recognizing m^6^A modifications in circRNAs. To date, studies of YTHDF1 have mainly focused on the promotion of mRNA translation. For example, Yang et al. [24] found that YTHDF1 could recognize and bind to m^6^A-modified lysosomal proteases to promote their translation. Tao Liu et al. [37] found that YTHDF1 promotes EIF3C translation in an m^6^A-dependent manner by binding to m^6^A-modified EIF3C mRNA. Unfortunately, none of these studies investigated the specific mechanism through which YTHDF1 promotes translation. Our experimental results showed that YTHDF1 could bind not only to m^6^A-modified mRNAs but also to m^6^A-modified circRNAs to promote ceRNA function. The specific mechanism, however, is unclear. Whether YTHDF1 functions as an architectural protein to facilitate the binding of circaALG1 to miR-342-5p or whether YTHDF1 needs to recruit other functional proteins to facilitate the binding of circaALG1 to miR-342-5p remains unclear. We look forward to solving this question in a follow-up study.

## Conclusions

**Fig. 8:**
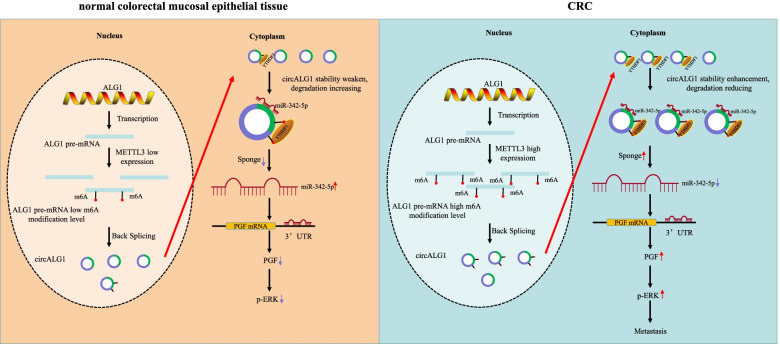
Experimental hypothesis: CircALG1 relieves the inhibitory effect of miR-342-5p on PGF mRNA expression by competitively binding to miR-342-5p and promotes PGF expression to result in enhanced invasion of CRC. The high expression of METTL3 in CRC increased the m6A modification level of ALG1 pre-mRNA, and then increased the m6A modification level of cirALG1. The m^6^A modification of circALG1 enhances its ability to bind to miR-342-5p and promotes circALG1 ceRNA action by enhancing its stability

Through a human circRNA microarray assay and bioinformatics analyses, we first found that circALG1 was highly expressed in both cancer tissues and peripheral blood from patients with CRC and that this high circALG1 expression promoted CRC metastasis through the miR-342-5p/PGF signalling axis. The m^6^A modification of circALG1 enhanced the binding ability of this circRNA to miR-342-5p and further promoted its ceRNA mechanism (Fig. 8). These experimental results suggested that circALG1 could be used as a potential target for early diagnosis and antitumour metastasis therapy. This study still has some limitations. Although circALG1 is highly expressed in the peripheral blood of patients with CRC, the sample size in this study was relatively small to identify it as a definitive target for early diagnosis. Due to a lack of follow-up assessments, the prognostic value of circALG1 remains unknown. The mechanism through which YTHDF1 mediates the binding of circALG1 to miR-342-5p remains unclear. These aspects will be the focus of our future research.

## Supplementary Information

**Additional file 1: Figure S1.** Detection of cell and tissue expression levels and transfection efficiency. **A.** SRAMP was used to predict the presence of an m^6^A modification in circALG1 and circCOL6A3. **B.** qRT-PCR detection of the circALG1 expression levels in 5 cell lines: FHC, HT29, HCT116, SW480, and SW620 cells. **C.** qRT-PCR detection of the efficiency of circALG1 overexpression in HCT116 cells. **D.** qRT-PCR detection of the efficiency of circALG1 interference in SW480 cells. The si-3 sequence, which exhibited the highest interference efficiency, was selected to construct the shRNA. The results are presented as the mean ± s.d. and are representative of at least 3 independent experiments. *p < 0.05, **p < 0.01, ***p < 0.001, ^#^*p *> 0.05.

**Additional file 2: Figure S2.** Parental linear ALG1 had no functional effect on circALG1. **A.** qRT-PCR detection of the expression of linear ALG1 mRNA after circALG1 overexpression or interference. **B.** qRT-PCR and WB assays were performed to detect the interference and overexpression efficiency of ALG1. ALG1 si-2 was selected for subsequent experiments. **C.** Representative images and bar graphs of Transwell migration and invasion assays of cells with different ALG1 expression levels in HCT116 and SW480 cells. The results are presented as the mean ± s.d. and are representative of at least 3 independent experiments. *p < 0.05, **p < 0.01, ***p < 0.001, ^#^*p *> 0.05.

**Additional file 3: Figure S3.** The m^6^A modification of circALG1 promoted CRC metastasis in HCT16 cells. **A.** MeRIP assays of m^6^A-modified circALG1 in HCT116 cells. **B.** qRT-PCR detection of the circALG1 expression level. **C.** MeRIP analysis of the level of m^6^A-modified circALG1. **D.** Representative images and bar graphs of Transwell migration and invasion assays of cells with different circALG1 m^6^A modification levels. The results are presented as the mean ± s.d. and are representative of at least 3 independent experiments. *p < 0.05, **p < 0.01, ***p < 0.001, ^#^*p *> 0.05.

**Additional file 4: Figure S4.** CircALG1/miR-342-5p/PGF axis contributed to CRC metastasis. **A.** qRT-PCR detection of the transfection efficiency of miRNA mimics. **B.** qRT-PCR detection of the miR-342-5p expression levels in tumours and adjacent tissues from patients with CRC. **C.** qRT-PCR detection of the transfection efficiency of the miR-342-5p inhibitor. **D.** Gene ontology (GO) enrichment analysis of differentially expressed genes after circALG1 silencing in SW480 cells. **E.** qRT-PCR and WB analyses were performed to assess the expression levels of PGF in FHC, HT29, HCT116, SW480, and SW620 cells. **E.** qRT-PCR and WB assays were performed to assess the overexpression efficiency of PGF in HCT116 cells. **F.** qRT-PCR and WB analyses were conducted to assess the interference efficiency of PGF in SW480 cells, and PGF si-1 was selected for functional experiments. The results are presented as the mean ± s.d. and are representative of at least 3 independent experiments. *p < 0.05, **p < 0.01, ***p < 0.001, ^#^*p *> 0.05.

**Additional file 5: Figure S5.** Determination of the interference efficiency. **A-B.** Representative images of Transwell migration and invasion assays of cells with different PGF expression levels in HCT116 and SW480 cells. **C. **qRT-PCR and WB assays were performed to detect the interference and overexpression efficiency of PGF in HCT116 circALG1 overexpression and SW480 sh-circALG1 cells. **D-E.** Representative images of Transwell migration and invasion assays of cells with PGF inhibitor and treatment with the circALG1 overexpression plasmid or miR-342-5p inhibitor (D) and cells with PGF overexpression and treatment with the circALG1 inhibitor or miR-342-5p mimics (E). The results are presented as the mean ± s.d. and are representative of at least 3 independent experiments. *p < 0.05, **p < 0.01, ***p <0.001, ^#^*p *> 0.05.

**Additional file 6: Figure S6.** M6A modification enhanced the stability of circALG1. **A.** qRT-PCR and WB assays were performed to detect the interference efficiency of YTHDF1 in SW480 cells, and YTHDF2 si-2 was selected for functional experiments. **B.** qRT-PCR and WB assays wereperformed to detect the interference efficiency of METTL3 in SW480 cells, and METTL3 si-2 was selected for functional experiments.** C. **CircALG1 stability in METTL3 si-NC/si-2 SW480 cells was determined by qRT-PCR after actinomycin D treatment for the time indicated. **D.** MeRIP analysis of ALG1 pre-mRNA m6A modification levels. **E. **qRT-PCR was performed to detect changes in circALG1 expression after METTL3 interference. The results are presented as the mean ± s.d. and are representative of at least 3 independent experiments. *p <0.05, **p < 0.01, ***p < 0.001, ^#^*p *> 0.05.

## Data Availability

The data and materials of this study are available from the corresponding author upon reasonable request.

## Abbreviations

ceRNA: Competitive endogenous RNA
circRNAs: Circular RNAs
CRC: Colorectal cancer
FBS: Foetal bovine serum
gDNA: Genomic DNA
HE: Haematoxylin and eosin
lncRNAs: Long noncoding RNAs
m^6^A: N^6^-methyladenosine
MeRIP: Methylated RNA immunoprecipitation
miRNAs: MicroRNAs
MREs: MiRNA response elements
PBS: Phosphate-buffered saline
PGF: Placental growth factor
qRT-PCR: Quantitative reverse-transcription polymerase chain reaction
RAP: RNA antisense purification
RIP: RNA-binding protein immunoprecipitation
SNPs: Single nucleotide polymorphisms
SPF: Specific pathogen-free
STRs: Short tandem repeats
