# Correction to: The N6-methyladenosine modification of circALG1 promotes the metastasis of colorectal cancer mediated by the miR-342-5p/PGF signalling pathway

**DOI:** 10.1186/s12943-022-01571-3

**Published:** 2022-04-20

**Authors:** Changwei Lin, Min Ma, Yi Zhang, Liang Li, Fei Long, Canbin Xie, Hua Xiao, Teng Liu, Buning Tian, Kaiyan Yang, Yihang Guo, Miao Chen, Jin Chou, Ni Gong, Xiaorong Li, Gui Hu

**Affiliations:** 1 Department of Gastrointestinal Surgery, The Third Xiangya Hospital of Central South University, Changsha, 410013 China; 2 Department of Hepatobiliary and Intestinal Surgery, Hunan Cancer Hospital and the Affiliated Cancer Hospital of Xiangya School of Medicine, Central South University, Changsha, 410013 China; 3 Hunan Chest Hospital, Changsha, 410013 China

**Correction to: Mol Cancer 21, 80 (2022)**

**https://doi.org/10.1186/s12943-022-01560-6**

Following the publication of the original article [1], the authors noticed that the supplementary files are outdated. Updated files are captured as supplementary files in this article.

The original article has been corrected.

## Supplementary Information

**Additional file 1: Figure S1.** Detection of cell and tissue expression levels and transfection efficiency. **A.** SRAMP was used to predict the presence of an m^6^A modification in circALG1 and circCOL6A3. **B.** qRT-PCR detection of the circALG1 expression levels in 5 cell lines: FHC, HT29, HCT116, SW480, and SW620 cells. **C.** qRT-PCR detection of the efficiency of circALG1 overexpression in HCT116 cells. **D.** qRT-PCR detection of the efficiency of circALG1 interference in SW480 cells. The si-3 sequence, which exhibited the highest interference efficiency, was selected to construct the shRNA. The results are presented as the mean ± s.d. and are representative of at least 3 independent experiments. *p < 0.05, **p < 0.01, ***p < 0.001, ^#^*p* > 0.05.

**Additional file 2: Figure S2.** Parental linear ALG1 had no functional effect on circALG1. **A.** qRT-PCR detection of the expression of linear ALG1 mRNA after circALG1 overexpression or interference**. B.** qRT-PCR and WB assays were performed to detect the interference and overexpression efficiency of ALG1. ALG1 si-2 was selected for subsequent experiments. **C.** Representative images and bar graphs of Transwell migration and invasion assays of cells with different ALG1 expression levels in HCT116 and SW480 cells. The results are presented as the mean ± s.d. and are representative of at least 3 independent experiments. *p < 0.05, **p < 0.01, ***p < 0.001, ^#^*p* > 0.05.

**Additional file 3: Figure S3.** The m^6^A modification of circALG1 promoted CRC metastasis in HCT16 cells. **A.** MeRIP assays of m^6^A-modified circALG1 in HCT116 cells. **B.** qRT-PCR detection of the circALG1 expression level. **C.** MeRIP analysis of the level of m^6^A-modified circALG1. **D.** Representative images and bar graphs of Transwell migration and invasion assays of cells with different circALG1 m^6^A modification levels. The results are presented as the mean ± s.d. and are representative of at least 3 independent experiments. *p < 0.05, **p < 0.01, ***p < 0.001, ^#^*p* > 0.05.

**Additional file 4: Figure S4.** CircALG1/miR-342-5p/PGF axis contributed to CRC metastasis. **A.** qRT-PCR detection of the transfection efficiency of miRNA mimics. **B.** qRT-PCR detection of the miR-342-5p expression levels in tumours and adjacent tissues from patients with CRC. **C.** qRT-PCR detection of the transfection efficiency of the miR-342-5p inhibitor. **D.** Gene ontology (GO) enrichment analysis of differentially expressed genes after circALG1 silencing in SW480 cells. **E.** qRT-PCR and WB analyses were performed to assess the expression levels of PGF in FHC, HT29, HCT116, SW480, and SW620 cells. **F.** qRT-PCR and WB assays were performed to assess the overexpression efficiency of PGF in HCT116 cells. **G** qRT-PCR and WB analyses were conducted to assess the interference efficiency of PGF in SW480 cells, and PGF si-1 was selected for functional experiments. The results are presented as the mean ± s.d. and are representative of at least 3 independent experiments. *p < 0.05, **p < 0.01, ***p < 0.001, ^#^*p* > 0.05.

**Additional file 5: Figure S5.** Determination of the interference efficiency. **A-B.** Representative images of Transwell migration and invasion assays of cells with different PGF expression levels in HCT116 and SW480 cells. **C.** qRT-PCR and WB assays were performed to detect the interference and overexpression efficiency of PGF in HCT116 circALG1 overexpression and SW480 sh-circALG1 cells. **D-E.** Representative images of Transwell migration and invasion assays of cells with PGF inhibitor and treatment with the circALG1 overexpression plasmid or miR-342-5p inhibitor (D) and cells with PGF overexpression and treatment with the circALG1 inhibitor or miR-342-5p mimics (E). The results are presented as the mean ± s.d. and are representative of at least 3 independent experiments. *p < 0.05, **p < 0.01, ***p < 0.001, ^#^*p* > 0.05.

**Additional file 6: Figure S6.** M6A modification enhanced the stability of circALG1. **A.** qRT-PCR and WB assays were performed to detect the interference efficiency of YTHDF1 in SW480 cells, and YTHDF2 si-2 was selected for functional experiments. **B.** qRT-PCR and WB assays were performed to detect the interference efficiency of METTL3 in SW480 cells, and METTL3 si-2 was selected for functional experiments. **C.** CircALG1 stability in METTL3 si-NC/si-2 SW480 cells was determined by qRT-PCR after actinomycin D treatment for the time indicated. **D.** MeRIP analysis of ALG1 pre-mRNA m6A modification levels. **E.** qRT-PCR was performed to detect changes in circALG1 expression after METTL3 interference. The results are presented as the mean ± s.d. and are representative of at least 3 independent experiments. *p < 0.05, **p < 0.01, ***p < 0.001, ^#^*p* > 0.05.
