# Supplementary material for: The N6-methyladenosine modification of circALG1 promotes the metastasis of colorectal cancer mediated by the miR-342-5p/PGF signalling pathway
Source: Mol Cancer. 2022 Mar 19;21:80. doi: 10.1186/s12943-022-01560-6 (PMC8933979; doi:10.1186/s12943-022-01560-6)
Supplement: Supplementary file 1 — Additional file 1: Figure S1. Detection of cell and tissue expression levels and transfection efficiency. A. SRAMP was used to predict the presence of an m6A modification in circALG1 and circCOL6A3. B. qRT-PCR detection of the circALG1 expression levels in 5 cell lines: FHC, HT29, HCT116, SW480, and SW620 cells. C. qRT-PCR detection of the efficiency of circALG1 overexpression in HCT116 cells. D. qRT-PCR detection of the efficiency of circALG1 interference in SW480 cells. The si-3 sequence, which exhibited the highest interference efficiency, was selected to construct the shRNA. The results are presented as the mean ± s.d. and are representative of at least 3 independent experiments. *p < 0.05, **p < 0.01, ***p < 0.001, #p > 0.05. [file 12943_2022_1560_MOESM1_ESM.pdf]

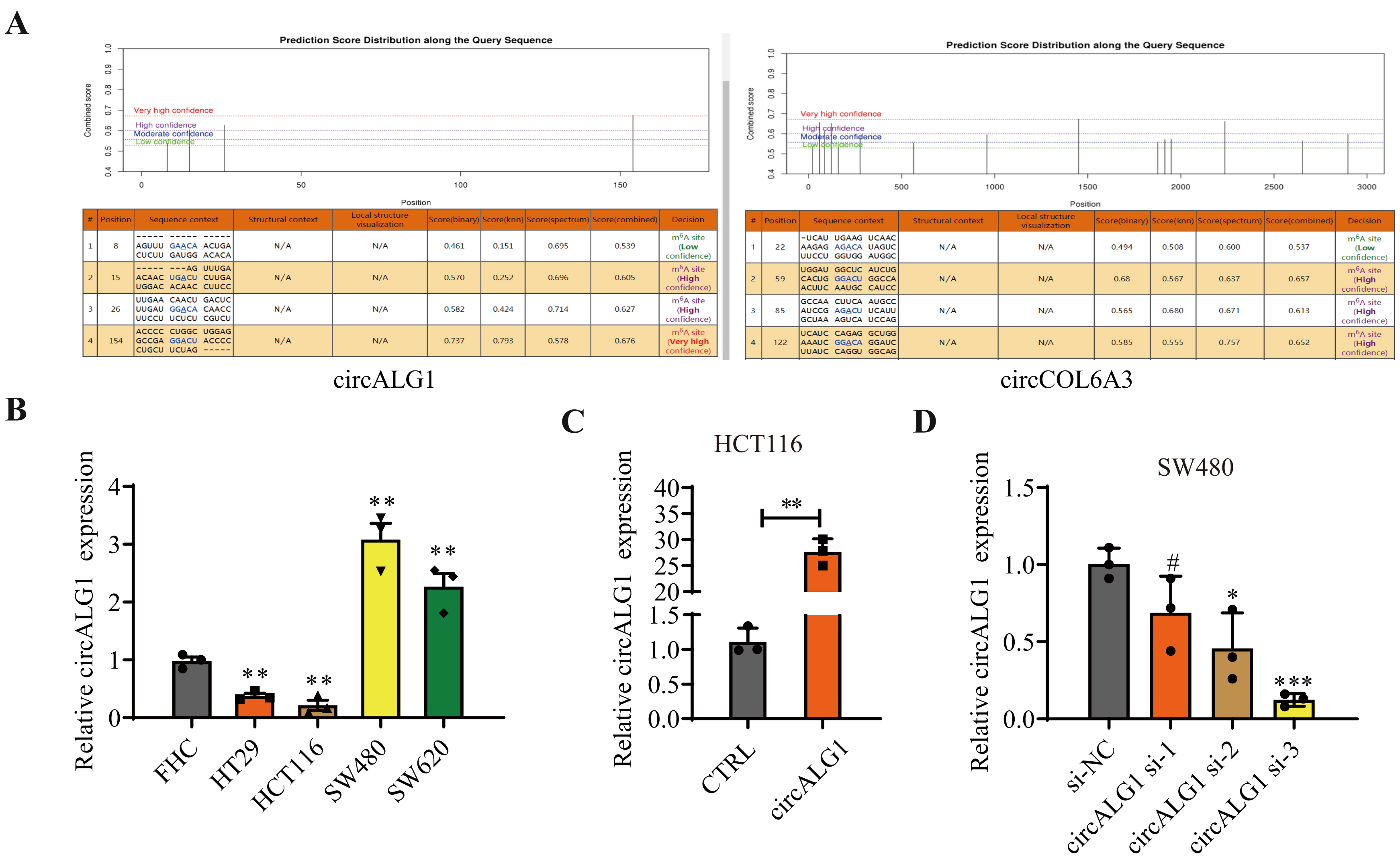

#	Position	Sequence context	Structural context	Local structure visualization	Score(binary)	Score(knn)	Score(spectrum)	Score(combined)	Decision
1	8	----- ----- -----<br>AGUUU GAACA ACUGA<br>CUCUU GAUGG ACACA	N/A	N/A	0.461	0.151	0.695	0.539	m6A site (Low confidence)
2	15	----- ---AG UUUGA<br>ACAAC UGACU CUUGA<br>UGGAC ACAAC CUUCC	N/A	N/A	0.570	0.252	0.696	0.605	m6A site (High confidence)
3	26	UUGAA CAACU GACUC<br>UUGAU GGACA CAACC<br>UUCCU UCUCU CGUCU	N/A	N/A	0.582	0.424	0.714	0.627	m6A site (High confidence)
4	154	ACCCC CUGGC UGGAG<br>GCCGA GGACU ACCCC<br>CUGCU UCUAG -----	N/A	N/A	0.737	0.793	0.578	0.676	m6A site (Very high confidence)

#	Position	Sequence context	Structural context	Local structure visualization	Score(binary)	Score(knn)	Score(spectrum)	Score(combined)	Decision
1	22	-UCAU UGAAG UCAAC<br>AAGAG AGACA UAGUC<br>UUCCU GGUGG AUGGC	N/A	N/A	0.494	0.508	0.600	0.537	m6A site (Low confidence)
2	59	UGGAU GGCUC AUCUG<br>CACUG GGACU GGCCA<br>ACUUC AAUGC CAUCC	N/A	N/A	0.68	0.567	0.637	0.657	m6A site (High confidence)
3	85	GCCAA CUUCA AUGCC<br>AUCCG AGACU UCAUU<br>GCUAA AGUCA UCCAG	N/A	N/A	0.565	0.680	0.671	0.613	m6A site (High confidence)
4	122	UCAUC CAGAG GCUGG<br>AAAUC GGACA GGAUC<br>UUAUC CAGGU GGCAG	N/A	N/A	0.585	0.555	0.757	0.652	m6A site (High confidence)
